# Modulation of rumen bacterial community and feed utilization in camel and sheep using combined supplementation of live yeast and microalgae

**DOI:** 10.1038/s41598-022-16988-5

**Published:** 2022-07-29

**Authors:** Alaa Emara Rabee, Boshra R. Younan, Khalid Z. Kewan, Ebrahim A. Sabra, Mebarek Lamara

**Affiliations:** 1 Animal and Poultry Nutrition Department, Desert Research Center, Cairo, Egypt; 2 Genetic Engineering and Biotechnology Research Institute, University of Sadat City, Sadat City, Egypt; 3 Forest Research Institute, University of Quebec in Abitibi-Temiscamingue, Rouyn-Noranda, Canada

**Keywords:** Antimicrobials, Applied microbiology, Bacteria, Microbial communities, Environmental microbiology, Immunology, Applied immunology

## Abstract

The combination of live yeast and microalgae as feed supplementation could improve rumen fermentation and animal productivity. This study aimed to investigate the impact of a mixture of (YA) yeast (*Saccharomyces cerevisiae*) and microalgae (*Spirulina platensis* and *Chlorella vulgaris*) as feed supplementation on feed intake, rumen disappearance of barley straw, bacteria, and fermentation, blood parameters of camels and sheep. Three fistulated camels and three fistulated rams were fed a concentrates mixture and ad libitum barley straw as a basal diet alone or supplemented with YA mixture. The dietary supplementation improved the feed intake, rumen disappearance of barley straw nutrients, and the blood immunity parameters. The YA supplementation affected rumen fermentation as well as the composition and diversity of rumen bacteria; however, the response to the supplementation varied according to animal species. Principle Coordinate Analysis (PCoA) separated bacterial communities based on animal species and feeding treatment. Phylum Bacteroidetes and Firmicutes dominated the bacterial community; and the dominant genera were *Prevotella, RC9_gut_group*, *Butyrivibrio*, *Ruminococcus*, *Saccharofermentans*, *Christensenellaceae*_R-7_group, and *Succiniclasticum.* Our results suggest positive impacts of YA supplementation in rumen fermentation and animal performance.

## Introduction

The low productivity of animals in the arid regions is attributed mainly to feed deficiency, wherever animal feeding relies on the low-protein and high-fiber roughages^1^. A huge amount of crop straw is generated globally every year^2^. Barley is grown in a wide range of adverse environmental conditions, including drought and soil salinity; thus, it is considered the main crop in desert regions^3^. Barley provides large quantities of lignocellulosic straw that could be used in the feeding of desert ruminants^4,5^. However, higher lignocellulose represents a barrier towards the efficient microbial fermentation of barley in the animal rumen^6^.

The fermentation in the rumen depends on the interactions between the microbial groups, consisting of bacteria, archaea, protozoa, and fungi^7^. Rumen microbiota convert the components of animal diets such as cellulose, hemicellulose, starch, and protein to microbial proteins and volatile fatty acids that provide the host animal with about 70% of daily protein and energy requirements^7,8^. Therefore, any modulations in the rumen microbiota and fermentation could have a significant impact on the physiology and efficiency of the host-animal. Rumen bacteria represent the majority of the microbial communities in the rumen, approximately 88% of microbial populations, with more than 10^10^cells /ml^7,9^. The composition of the animal diet and animal species are the main drivers of the composition of rumen microbial groups^8,9^. Understanding the alterations of the rumen microbial ecosystem in different animal species and under different dietary supplementation offers the possibility to design suitable feeding strategies to optimize the rumen microbial fermentation, including fiber digestion of desert ruminants^7–9^. Desert dwelling ruminants such as camels and Barki sheep contribute significantly to food security in arid countries by providing milk and meat under harsh conditions^10^. These animals have been adapted to live in adverse desert conditions, including heat stress, scarcity of water, and availability of poor-quality feedstuff^11^. Camel is multipurpose animal that is adapted to desert harsh conditions by the unique feeding behavior, physiological mechanisms, and the functional structure of the digestive tract^12^. Barki sheep is the main sheep breed in the arid regions in North Africa and the Middle East, as this breed is well adapted to desert conditions^13^. The differences in the anatomy and physiology of the digestive tract as well as feeding behavior between camels and other ruminants resulted in differences in the feeding requirements, rumen microbial fermentation, and metabolism^14^. Gihad et al.^15^ indicated that camels have high efficiency than sheep in dry matter and crude fiber digestion, and nitrogen utilization. Moreover, a comparative study on camels and sheep^16^ showed some discrepancies in the composition of the rumen bacterial community; for instance, camels showed higher *Ruminococcus* and *Butyrivibrio*, and *Prevotella*, while sheep have higher *RC9_gut_group*. Therefore, the response of animal species to dietary intervention could be varied^17^.

Furthermore, intensification of animal production and restriction of antibiotics use in animal production are the main drivers of the transition to the safe and novel feed additives^18,19^. Probiotic and prebiotic feed additives or their combinations have gained interest to manipulating rumen fermentation^20,21^. However, feed additives need to be studied continuously to examine their efficacy, which depends on the nature of compounds, the composition of animal diet, and the response of the rumen microbiome^22^. Supplementation of the animal diet with probiotics such as live yeast (*Saccharomyces cerevisiae*) alone or with phytogenic substrates has been shown to affect the rumen microbiome^20,22^. Previous studies^23,24^ indicated that the supplementation of the cattle and camel with live yeast improved the total VFA production and stabilized rumen pH, and affected the abundance of some bacterial groups, including fibrolytic bacteria. Moreover, in recent years, algae or their extracts have gained interest as feed additives in livestock diets^19^.

*Chlorella* and *Spirulina* are well-known microalgae worldwide; they are rich sources of proteins, essential amino acids, vitamins, pigments, fatty acids, minerals, and other natural bioactive compounds^25,26^. Therefore, microalgae supplementation can boost the rumen microbiota to enhance diet fermentation. Previous studies indicated that microalgae feed additives improved animal health, growth, and fertility^27^. Tsiplakou et al.^28^ reported that the inclusion of *Chlorella* in goats’ diet affected the abundance of rumen bacteria and milk fatty acids profile. The availability of polysaccharides and other bioactive compounds is the main reason to use of microalgae or their derivatives as a prebiotic to promote gut health and modulate gut microbiota composition^29^. Therefore, it was hypothesized that using mixtures of prebiotics such as microalgae, and probiotics such as live yeast, as feed additives is an effective strategy to improve animal health and productivity by promoting the activities of rumen microbiota to enhance rumen fermentation and stimulate the immunity^21,30,31^. Grimm et al.^21^ supplemented the horses with a combination of live yeast and microalgae (*Aurantiochytrium limacinum*) and found that the supplementation affected the abundance of some fibrolytic gut bacteria.

Very limited information is available regarding the supplementation of the combination of live yeast and microalgae to animal diet. This study aimed to determine the effect of a mixture of *S. cerevisiae* and microalgae mixture (*Spirulina platensis* and *Chlorella vulgaris)* on feed intake, rumen fermentation and bacteria, blood parameters, and ruminal disappearance of dry matter (DM), crude protein (CP), and neutral detergent fiber (NDF) of barley straw in two different animal species, camels and sheep.

## Methods

### Animals and ethics

The present experiment was conducted at Maryoout Research Station, Desert Research Center, Alexandria, Egypt. Three fistulated adult camels (*Camelus dromedaries,* Maghraby breed; age, 6 years; average body weight, 450 ± 5.5 kg) and three fistulated adult rams (*Ovis aries*, Barki breed; age, 5 years; average body weight, 47 ± 1.7 kg) were assigned to receive two dietary treatments successively. This study was conducted under guidelines set by the Department of Animal and Poultry Production, Desert Research Center, Egypt. Moreover, the project was approved by the Institutional Animal Care and Use Committee, Faculty of Veterinary Medicine, University of Sadat City, Egypt (Project Reference: VUSC00008). All methods were performed in compliance with the ARRIVE guidelines. In addition, the project does not include euthanasia of the experimental animals. The sample size was decided based on the availability of similar animals with similar physical and physiological status.

### Experimental diets

Throughout the experiment, all animals were offered ad libitum barley straw. Moreover, concentrates mixture was offered to camels at 1400 g/head and rams at 450 g/head. The concentrates mixture was either unsupplemented (control) or supplemented with yeast and algae combination (YA) (25% *S. cerevisiae*, 50% *S. platensis,* and 25% *C. vulgaris*). The YA combination was offered gradually and was supplied to the camels at 48 g/head and for sheep at 12 g/head, which represented 1% of daily total dry matter intake. The experiment was conducted for two periods, in the first period, the animals were offered the control diet for 21 days before the sampling period. Subsequently, all animals were switched to the second period with the supplemented concentrate mixture for 21 days before the sampling period. Thus, the experimental groups were (1) camels fed the control diet without supplementation (CC); (2) Rams fed the control diet (CR); (3) camels supplemented with YA mixture (TC); and (4) rams supplemented by YA mixture (TR). All animals were housed individually in well-ventilated pens and offered free drinking water and received the experimental diet as per treatment twice daily at 08:00 and 14:00 h. All refused feed was quantified to determine the feed intake. Concentrate feed mixture consisted of corn 57.5%, soybean meal 23%, wheat bran 19%, limestone 2.5%, salt 1.5%, sodium bicarbonate 0.5%, premix 0.4%, and antitoxins 0.1%. The chemical analysis of barley straw and concentrates feed mixtures are presented in Table 1. After the adaptation period, the disappearances of dry matter (DM), crude protein (CP), and neutral detergent fiber (NDF) of barley straw were conducted.

**Table 1:** The chemical composition (%) of control (C) and treatment (T) concentrates mixtures and barely straw.

	% DM	% OM	EE %	CP%	NDF %	Ash %
Barely straw	88	77.2	3.1	4.75	67	10.8
Control concentrates (C)	90	79.5	4.5	14.5	53	10.5
Treatment concentrates (T)	91	82	5.4	17.5	46	9

*DM* dry matter, *CP* crude protein, *EE* ether extract, *NDF* neutral detergent fiber.

### Rumen disappearance of barley straw

Barley straw was oven-dried at 50 °C and then ground to pass a 1 mm sieve; then a 2.5 g was weighted into nylon bags (10 × 20 cm; pore size = 50 μm). One nylon bags was prepared for each of four times for each animal (3, 6, 12, 48 h). A total of four bags were placed into the rumen of each camel or ram before morning feeding. The bags were retrieved from the animal rumen after 3, 6, 12, 48 h. After the removal from the rumen, the bags were rinsed with water until the water ran clear; then squeezed and dried at 60 °C for 48 h to determine the disappearance of dry matter (DMD), neutral detergent fiber (NDFD), and crude protein (CPD) of barley straw when animal fed the control diet (C) and treatment diet (YA).

### Rumen and blood samples

At the end of every experimental period, rumen and blood samples were collected before morning feeding. Rumen contents were collected from the animal’s fistula before feeding and strained by two layers of cheesecloth. The rumen pH was recorded using a digital pH meter (WPA CD70). The rumen liquid was used in the analysis of rumen fermentation parameters and DNA isolation. Blood samples were collected from the jugular vein, and serum was separated by centrifuging at 10,000× g for 5 min and then was frozen for further analysis.

### Chemical analysis

The live yeast was a commercial product containing *S. cerevisiae* at the concentration of 2 × 10^7^ colony-forming units (CFU)/g. All the *Chlorella* and *Spirulina* that were used in the experiment were obtained in the form of green dried powder from Algal Biotechnology Unit, National Research Centre, Cairo, Egypt, and their chemical composition was previously reported in El-Sayed and El-Sheekh; and El-Feky et al.^32,33^. The contents of dry matter (DM), neutral detergent fiber (NDF), and crude protein (CP) were determined in experimental diets and barley straw before and after incubation in the rumen. DM was measured by drying the material for 48 h at 60 °C. NDF was determined by the method of Van Soest et al.^34^ without sodium sulfite. CP was determined according to AOAC^35^. The rumen ammonia (NH_3_-N) and total VFA concentrations were determined by steam distillation in Kjeldahl distillation equipment^35,36^. In addition, individual VFAs were measured using high-performance liquid chromatography (HPLC) and the VFA were separated using C18 column with a mobile phase containing 0.3% phosphoric acid^37^. Serum metabolites were analyzed after thawing using a spectrophotometer with commercial reagents (Biodiagnostic-diagnostic and research reagents, Giza, Egypt) according to manufacturer’s recommendations and immunoglobulin IgA, IgG, and IgM were determined using Enzyme-Linked Immunosorbent Assay (ELISA).

### Bacterial community analysis

#### DNA extraction, PCR amplification, and sequencing

One milliliter of every rumen sample was centrifuged at 13,000 rpm, and the remained precipitate was used for DNA extraction by i-genomic Stool DNA Extraction Mini Kit (iNtRON Biotechnology, Inc.) according to the manufacturer’s instructions. DNA was eluted in 50µL elution buffer, and DNA quality and quantity were checked by agarose gel electrophoresis and Nanodrop spectrophotometer, respectively. The V4 region of the bacterial 16S rDNA gene was amplified using 515F and 926R primers^38^. PCR amplification was conducted under the following conditions: 94 °C for 3 min; 35 cycles of 94 °C for 45 s, 50 °C for 60 s, and 72 °C for 90 s; and 72 °C for 10 min. PCR products purification, preparation for sequencing using Illumina MiSeq system were conducted according to the protocol described by Comeau et al.^39^ in Integrated Microbiome Resource (IMR, Dalhousie University, Halifax, NS, Canada). Briefly, PCR-amplicons were cleaned up and normalized using the high-throughput Invitrogen SequalPrep 96-well plate kit. Then, the samples were finally pooled to make one library for the sequencing.

#### Determination of copy number of bacterial 16S rDNA by using qPCR

Quantitative real-time PCR (qPCR) was used to determine the total bacterial 16S rDNA copy number in the rumen samples. Standards were generated using serial dilutions of DNA isolated from *Prevotella sp, Ruminococcus albus, Butyrivibrio hungatei* purchased from DSMZ (Braunschweig, Germany). Serial dilutions of the standards ranging from 10^1^ to 10^6^ copies of the 16S rDNA gene were used. The qPCR was performed using the Applied Biosystems StepOne system (Applied Biosystems, Foster City, USA). Bacterial primers F (5′-CGGCAACGAGCGCAACCC-3′) and R (5′-CCATTGTAGCACGTGTGTAGCC-3′)^40^ were applied. The 10-μL reaction consisted of 1 μL genomic DNA, 1 μL of each primer, and 7 μL SYBER Green qPCR- master mix (iNtRON Biotechnology, Inc.). The PCR conditions were as follows 40 cycles of 95 °C for 15 s and 60 °C for 60 s. The linear relationship between the threshold amplification (Ct) and the logarithm of 16S rDNA copy numbers of the standards was used to calculate the copy numbers of rumen bacteria per μL of DNA.

### Bioinformatics analysis

All the paired-end (PE) Illumina raw sequences were processed in R (version 3.5.2) using the DADA2 (version 1.11.3) pipeline as described by Callahan et al.^41^. Paired-end fastq files were demultiplexed and quality checks of forward and reverse reads were conducted based on the quality scores. Then sequence reads were quality filtered, trimmed, and dereplicated followed by merging the forward and reverse reads together to obtain the full denoised sequences. The reads were inspected for chimeras that were removed, and Amplicon Sequence Variants (ASVs) were generated. Taxonomic assignment of sequence variants was performed using a combination of the functions assign Taxonomy and assignSpecies and was compared using the SILVA reference database. Various alpha diversity indices, Chao1, Shannon, and InvSimpson were obtained. Beta diversity was assessed as the principal coordinate analysis (PCoA) based on bray–curtis dissimilarity.

### Statistical analysis

The results of the relative abundance of bacteria were tested for normality using the Shapiro–Wilk test, and non-normal values were then arcsine transformed. The effect of animal species (A), feeding treatment (T), and A × T interactions on the differences in feed intake, rumen fermentation parameters, bacterial copy numbers, blood biochemical and immunological parameters, microbial diversity, and relative abundances of bacterial groups were studied by Mixed ANOVA model using Repeated Measures function in IBM SPSS software v. 20.0^42^. The between-subjects factor was the animal species, and the within-subjects factor was feeding treatment. The Duncan test through One-Way ANOVA was carried out for mean separation between all experimental groups. For all statistical tests, a *P* < 0.05 was used as a threshold of statistical significance. Pearson correlation analysis was used to identify correlation relationships between feed intake, rumen fermentation parameters, blood parameters, nutrients disappearance, and relative abundances of bacterial genera. The correlation scores were visualized as a heatmap using PAST software^43^. All the sequences were deposited to the sequence read archive (SRA) under the accession number: PRJNA767400.

## Results

### Feed intake

The chemical compositions of barley straw and concentrates mixtures are presented in Table 1. The data shows that barley straw has lower crude protein (CP) and higher neutral detergent fiber (NDF) than concentrate mixtures. Additionally, concentrates mixtures of treatment (T) had higher CP and lower NDF compared to the control concentrates mixture (C). The feed intake results expressed as g/ Kg^0.75^ (Kilogram metabolic body weight) are described in Table 2. The inclusion of YA mixture in animals’ diets increased the feed intake significantly (Table 2). Furthermore, species' feed intake was affected significantly, and the interaction between animal species and treatment was significant (*P* < 0.05). Camel group TC showed higher dry matter intake (DMI), organic matter intake (OMI), crude protein intake (CPI) from roughage, and neutral detergent fiber feed intake (NDFI). In contrast, sheep group TR showed a higher total CPI (Table 2).

**Table 2:** Effect of YA supplementation on roughage and total feed intake expressed as g/Kg ^0.75^ of camel and sheep.

Feed intake	Control (C)				Treatment (T)				*P* values		
	Camel (CC)		Sheep (CR)		Camel (TC)		Sheep (TR)		A	T	A × T
	Mean	SE	Mean	SE	Mean	SE	Mean	SE			
**Roughage feed intake**											
DMI g/kg ^0.75^	33.1^c^	0.5	14.5^a^	1.6	37.2^d^	0.3	25.6^b^	1.8	< 0.0001	0.004	0.052
OMI g/kg ^0.75^	29.03^c^	0.46	12.72^a^	1.42	32.65^c^	0.28	22.42^b^	1.59	< 0.0001	0.004	0.052
CPI g/Kg ^0.75^	1.6^c^	0.02	0.7^a^	0.07	1.8^d^	0.015	1.2^b^	0.08	< 0.0001	0.004	0.052
NDFI g/Kg ^0.75^	22.2^c^	0.35	9.7^a^	1.1	25^d^	0.2	17.1^b^	1.2	< 0.0001	0.004	0.052
**Total feed intake**											
DMI g/kg ^0.75^	46.2^b^	0.5	37^a^	1.6	50.5^b^	0.3	48.3^b^	1.8	0.01	0.003	0.052
OMI g/kg ^0.75^	40.67^b^	0.46	32.59^a^	1.42	44.66^b^	0.28	42.92^b^	1.59	0.011	0.003	0.046
CPI g/Kg ^0.75^	3.5^a^	0.02	3.95^b^	0.08	4.1^b^	0.01	5.15^c^	0.08	< 0.0001	< 0.0001	0.008
NDFI g/Kg ^0.75^	29.2^c^	0.35	21.6^a^	1.1	31.1^c^	0.2	25.8^b^	1.2	0.002	0.026	0.26

A, animal species; T, feeding treatment; AXT, interaction between animal species and feeding treatment.

*DMI* dry matter intake, *OMI* Organic matter intake, *CPI* crude protein intake, *NDFI* neutral detergent fiber intake, *SE* standard error, *SEM* standard error of the mean.

^a,b,c,d^: Means in the same row with different superscripts differ significantly (*P* < 0.05).

### Rumen disappearance of nutrients

The disappearances of barley nutrients (DM, CP, and NDF) at 3, 6, 12, 48 h are described in Table 3. The inclusion of the YA mixture in animals’ diets improved the disappearance of DM, CP, and NDF in the rumen of camel and sheep with a significant difference at 48 h (*P* < 0.05); and animal species did not affect the disappearance. Supplemented camel group (TC) showed the highest DMD at 48 h. Moreover, supplemented sheep group (TR) showed higher NDFD at all incubation times except for 48 h, whenever TC group showed the highest NDFD. Also, sheep group TR showed greater CPD at 48 h.

**Table 3:** Disappearance of dry matter (DMD %), crude protein (CPD %), neutral detergent fiber (NDFD%) of barley straw at 3, 6, 12 and 48 h of incubation for in the rumen of supplemented and non-supplemented camels and sheep.

	Control (C)				Treatment (T)				*P* values		
	Camel (CC)		Sheep (CR)		Camel (TC)		Sheep (TR)		A	T	A × T
	Mean	SE	Mean	SE	Mean	SE	Mean	SE			
**DMD%**											
3 h	19.5	0.9	18.2	1.0	21.0	0.5	21.0	1.0	0.49	0.07	0.52
6 h	20.2	0.75	19.4	0.77	22	0.7	20.2	1.7	0.16	0.42	0.76
12 h	29.3	3.9	34	1.9	31.7	1.7	33.7	1.5	0.28	0.75	0.717
48 h	46.5^a^	1.6	44.9^a^	3.7	53.1^b^	0.4	49.5^b^	2.8	0.46	0.009	0.18
**CPD%**											
3 h	12.4	2.0	12.8	0.7	15.7	0.6	15.0	0.2	0.9	0.07	0.6
6 h	15.24	2.2	15.2	0.8	18.4	0.5	17	1	0.62	0.08	0.54
12 h	25.8	4.7	20.7	1	28.05	1.8	30.2	2.2	0.67	0.06	0.18
48 h	40.8^ab^	5.8	32.4^a^	0.6	47.4^b^	3.4	50.3^b^	1.5	0.48	0.02	0.17
**NDFD%**											
3 h	4.1^a^	1.1	5.3^a^	0.7	5.8^ab^	0.1	8.4^b^	1.1	0.14	0.02	0.34
6 h	5.6	1	7.2	0.4	7.6	0.6	9.2	2	0.13	0.25	0.9
12 h	16.25	3.1	21.7	4.9	18.4	1.96	23.3	1.2	0.14	0.60	0.94
48 h	39.1^a^	2.05	37.2^a^	3.7	45.8^b^	0.34	42.5^b^	3.1	0.50	0.006	0.55

^a,b^: Means in the same row with different superscripts differ significantly (*P* < 0.05).

### Rumen fermentation parameters

The effect of the inclusion of YA supplementation on rumen fermentation parameters is shown in Table 4. Neither YA supplementation nor animal species affected rumen pH. Animal species affected rumen ammonia and total VFA concentration significantly (*P* < 0.05) (Table 4). The supplementation of the YA mixture affected the molar proportions of acetic, propionic, and isobutyric acids significantly (*P* < 0.05). For example, the molar proportions of propionic was increased in supplemented sheep (TR) and decreased in the supplemented camel (TC). The molar proportion of isobutyric was increased in camels and sheep by YA supplementation. Additionally, the bacterial population was affected by YA supplementation and animal species (*P* < 0.05) as it was increased in supplemented camels (TC).

**Table 4:** Effect of YA supplementation on rumen fermentations parameters and bacterial population (Log10 copies/μL DNA) in the rumen of camels and sheep.

	Control (C)				Treatment (T)				*P* values		
	Camel (CC)		Sheep (CR)		Camel (TC)		Sheep (TR)		A	T	AxT
	Mean	SE	Mean	SE	Mean	SE	Mean	SE			
pH	6.6	0.06	6.4	0.24	6.3	0	6.4	0.1	0.8	0.07	0.17
Ammonia, mg/dl	10.3^a^	1.7	16.3^ab^	4	13.06^ab^	0.9	19.1^b^	2.5	0.02	0.42	0.1
VFA, meq/dl	13^c^	0.8	8.9^ab^	0.3	10.2^b^	0.3	8^a^	0.4	0.04	0.002	0.12
Acetic, %	63^b^	0.6	69.4^b^	0.4	65.2^b^	1.3	52.7^a^	4.2	0.24	0.03	0.01
Propionic, %	26.2^b^	0.56	19.6^a^	0.3	21.4^ab^	0.8	36.8^c^	3.6	0.06	0.04	0.006
Butyric, %	10.1^b^	0.1	9.8^b^	0.4	10^b^	0	8.2^a^	0.7	0.1	0.08	0.1
isobutyric, %	0.73^a^	0.08	1.1^a^	0.25	3.4^b^	0.63	2.3^b^	0.15	0.36	0.006	0.12
Bacteria population*	7.6^a^	0.08	7.25^a^	0.07	8.1^b^	0.1	7.5^a^	0.2	0.03	0.02	0.26

Bacteria population = Log 10 of 16S rDNA copy number / µL DNA.

^a,b,c^: Means in the same row with different superscripts differ significantly (*P* < 0.05).

### Blood parameters

The effect of YA supplementation and animal species on serum biochemical parameters and serum immunoglobulins IgA, IgG, and IgM are presented in Table 5. Animal species and YA supplementation affected the concentration of most of the serum biochemical parameters and serum immunoglobulins (*P* < 0.05). Higher IgA was observed in camel group CC; and camel group TC showed higher glucose, triglycerides, IgG, and IgM. Moreover, sheep group TR showed higher total protein.

**Table 5:** Effect of YA supplementation on the blood metabolic profile and immunological response (mg/dl) in camels and sheep.

	Control				Treatment				*P* values		
	Camel (CC)		Sheep (CR)		Camel (TC)		Sheep (TR)		A	T	AxT
	Mean	SE	Mean	SE	Mean	SE	Mean	SE			
Glucose, mg/dl	72^b^	2	45.33^a^	0.88	92.33^c^	11.33	45^a^	3.38	0.002	0.001	0.18
Total Protein, g/dl	6.1^a^	0.23	6.7^a^	0.15	6.3^a^	0.05	7.12^b^	0.23	0.002	0.02	0.66
Albumin, g/dl	4.1^b^	0.05	3.06^a^	0.20	3.8^b^	0.28	3.4^a^	0.2	0.042	0.83	0.12
Urea, mg/dl	26.7^a^	1.2	36^b^	3. 8	22.3^a^	1.85	29^ab^	2	0.024	0.09	0.06
Triglycerides, mg/dl	42.3^c^	1.8	14.7^a^	0.33	43.3^c^	3.3	27.7^b^	2.02	< 0.0001	0.048	0.07
Total lipids, mg/dl	148.8	5.5	141.63	9.24	156.51	17.41	147.50	3.06	0.55	0.41	0.90
IgG, mg/dl	228^c^	6.24	102.8^a^	3	235.75^c^	2.8	139.3^b^	7.13	0.0001	0.001	0.007
IgM, mg/dl	42.7^b^	2.02	19.5^a^	3.4	52.96^c^	1.21	35.84^b^	1.7	0.0001	0.007	0.31
IgA, mg/dl	30^c^	0.57	18.25^a^	0.63	25.6^b^	0.81	26.13^b^	1.23	0.007	0.023	0.0001

*IgG* Immunoglobulin G, *IgM* Immunoglobulin M, *IgA* Immunoglobulin A.

^a,b,c^: Means in the same row with different superscripts differ significantly (*P* < 0.05).

### Bacterial diversity and composition

**Figure 1:**
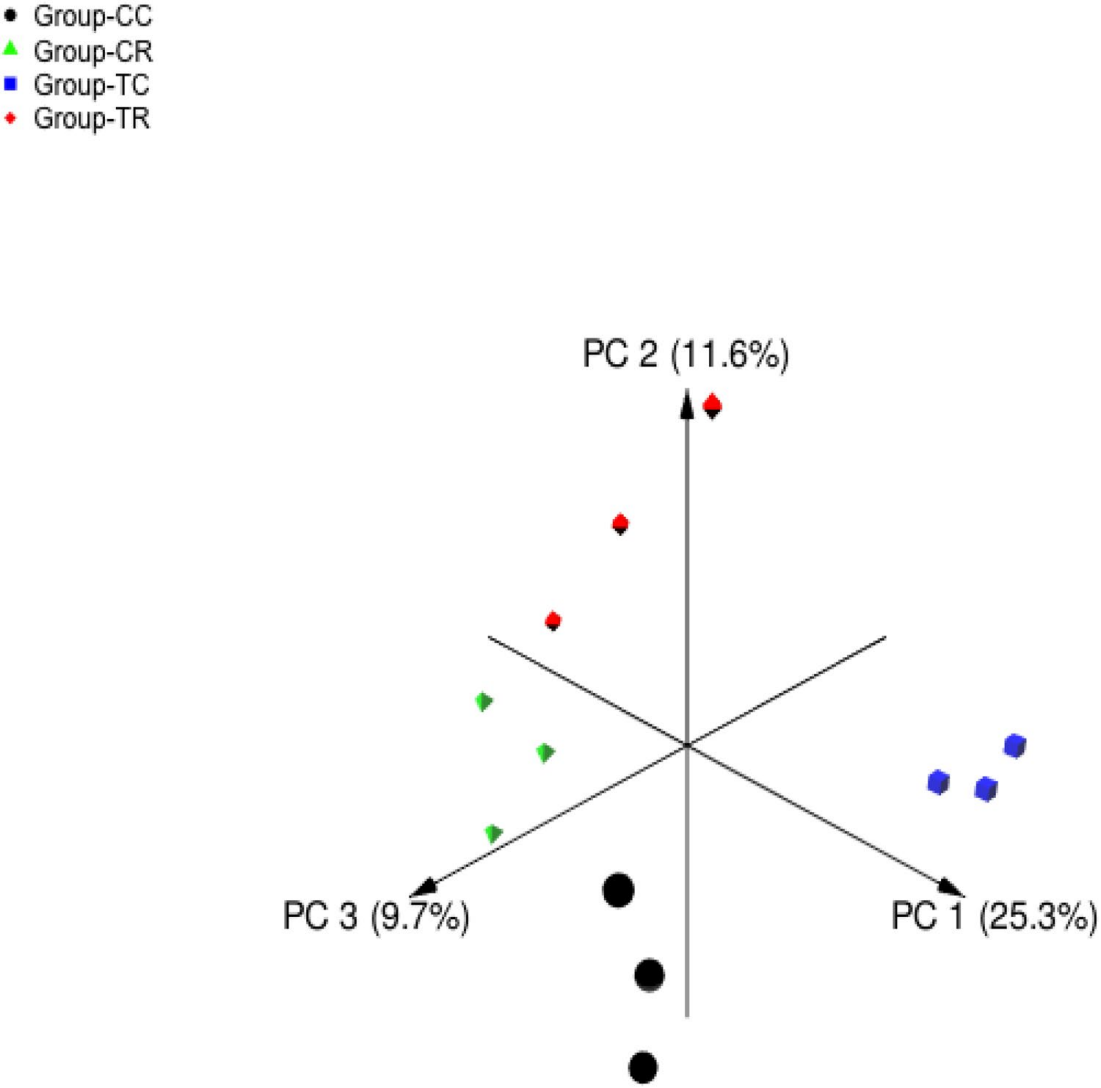
Principal coordinates analysis of bacterial communities in the rumen of supplemented and non-supplemented camels and sheep based on Bray–Curtis dissimilarity. The analysis was conducted between four experimental groups: black circles for non-supplemented camels, green triangles for non-supplemented sheep, blue squares for supplemented camels, and red triangles for supplemented sheep.

The sequencing of the V4 region on the 16S rDNA gene in 12 rumen samples resulted in 393 690 high-quality sequence reads with an average of 32 807 sequences per sample. Table 6 presents the effect of animal species and YA supplementation on the alpha diversity metrics. The results showed that feeding treatment and animal species affected the microbial diversity indices significantly (*P* < 0.05). Chao1 index was increased in camel group TC compared to CC and was declined in sheep group TR compared to CR. Shannon and InvSimpson indices were increased by YA supplementation. Principle Coordinate Analysis (PCoA) based on Bray Curtis dissimilarity metrics showed that bacterial communities of CC, CR, TC, and TR groups were separated distinctly by feeding treatment and animal species (Fig. 1).

**Table 6:** Averages of ASVs number, Chao1, Shannon, and Inverse Simpson indices of microbial communities in the rumen of camel and sheep supplemented and non-supplemented with YA mixture.

	Control				Treatment				*P* value		
	Camel (CC)		Sheep (CR)		Camel (TC)		Sheep (TR)		A	T	A × T
	Mean	SE	Mean	SE	Mean	SE	Mean	SE			
Chao1	453.85^a^	5.3	1408.4^c^	46.7	720.6^b^	60.15	602.43^ab^	112.3	0.002	0.025	0.002
Shannon	5.9^a^	0.05	6.34^b^	0.14	6.35^b^	0.11	6.6^b^	0.16	0.003	0.023	0.56
InvSimpson	283.9^a^	0.9	250.06^a^	33.04	447.75^b^	39	541.13^b^	32.75	0.327	0.002	0.131

*NS* Non-significance at *P* < 0.05.

^a,b,c^: Means in the same row with different superscripts differ significantly (*P* < 0.05).

**Figure 2:**
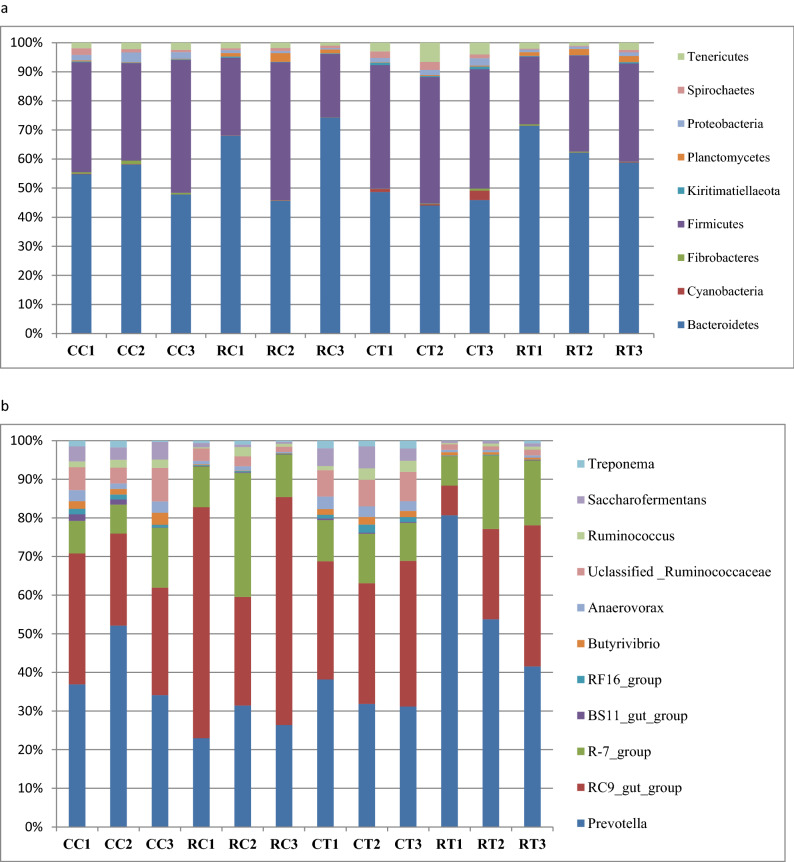
Relative abundance of bacterial families and genera. The relative abundances of dominant bacterial phyla (**a**) and bacterial genera (**b**) in the rumen of non-supplemented camels (CC1–CC3), non-supplemented sheep (CR1–CR3), supplemented camels (TC1–TC3), and supplemented sheep (TR1–TR3).

The taxonomic analysis showed that the bacterial community was assigned to 17 bacterial phyla. The bacterial community was predominated by phylum Bacteroidetes and Firmicutes. Other bacterial phyla that represented more than 1% were Planctomycetes, Proteobacteria, Spirochaetes, and Tenericutes. Furthermore, bacterial phyla that were found in less than 1% were Cyanobacteria, Fibrobacteres, and Kiritimatiellaeota (Table 7). Some phyla were observed exclusively in a specific group, including Actinobacteria and Synergistia, which were found in CR and TC groups. Phylum Chloroflexi was found only in CR and TR. Phylum Verrucomicrobia was observed in CC and TC whereas, phylum Elusimicrobia was observed in TC and TR groups. Phylum Lentisphaerae was not detected in TR, and phylum Patescibacteria was found only in the TC group. The results showed that the inclusion of YA mixture in animals’ diets affected the relative abundance of some bacterial phyla significantly (*P* < 0.05) (Table 7; Fig. 2a).

**Table 7:** Effect of YA supplementation on the average of relative abundance (%) of bacterial phyla in the rumen of camels and sheep under investigation.

Phylum	Control (C)				Treatment (T)				*P* value		
	Camel(CC)		Sheep (CR)		Camel (TC)		Sheep (TR)		A	T	AxT
	Mean	SE	Mean	SE	Mean	SE	Mean	SE			
Actinobacteria			0.6		0.06				ND	ND	ND
Bacteroidetes	53.4	3.04	62.2	8.7	45.5	1.32	63.9	3.77	0.055	0.56	0.39
Chloroflexi			0.047				0.12		ND	ND	ND
Cyanobacteria	0.12^a^	0.05	0.06^a^	0.02	1.5^b^	0.8	0.10^a^	0.02	0.013	0.034	0.051
Elusimicrobia					0.48		0.06		ND	ND	ND
Fibrobacteres	0.7	0.2	0.09	0.005	0.35	0.17	0.3	0.09	0.051	0.66	0.16
Firmicutes	38.8	3.5	31.6	7.7	41.7	0.8	29.9	3.37	0.12	0.90	0.62
Kiritimatiellaeota	0.22^a^	0.06	0.27^a^	0.081	0.68^b^	0.14	0.28^a^	0.11	0.228	0.048	0.051
Lentisphaerae	0.12	0.04	0.02	0.01	0.2	0.025	0	0	ND	ND	ND
NA	0.11^ab^	0.05	0.02^a^	0.008	0.23^b^	0.05	0.13^ab^	0.03	0.167	0.0013	0.896
Patescibacteria					0.13				ND	ND	ND
Planctomycetes	0.24^a^	0.06	1.8^b^	0.55	0.28^a^	0.03	1.8^b^	0.28	0.015	0.917	0.955
Proteobacteria	2.5^c^	0.44	0.7^a^	0.12	1.89^bc^	0.29	0.97^ab^	0.15	0.0018	0.552	0.198
Spirochaetes	1.38^ab^	0.45	0.85^a^	0.10	2.07^b^	0.4	0.45^a^	0.17	0.043	0.63	0.11
Synergistia			0.02		0.12				ND	ND	ND
Tenericutes	2.18^a^	0.16	1.55^a^	0.29	4.5^b^	1.07	1.9^a^	0.4	0.053	0.03	0.181
Verrucomicrobia	0.07				0.57				ND	ND	ND

*ND* Non-determined, *NA* Nonclassified phylum.

^a,b,c^: Means in the same row with different superscripts differ significantly (*P* < 0.05).

Phylum Bacteroidetes was not affected by feeding treatment and animal species. The members of this phylum were assigned to six families, BS11_gut_group, RF16_group, F082, Muribaculaceae, Prevotellaceae, and Rikenellaceae (Table 8). The members of the family Prevotellaceae and Rikenellaceae represented the majority of Bacteroidetes. Family Prevotellaceae was dominated by genus *Prevotella* that was affected significantly (*P* < 0.05) by YA supplementation, whenever it was declined in camel group TC compared to CC and was increased in sheep group TR compared to CR*.* Family Rikenellaceae was dominated by genus *RC9_gut_group* (Table 8 and Fig. 2).

**Table 8:** Effect of YA supplementation on the average of relative abundance (%) of dominant bacterial families and genera in the rumen of camels and sheep under investigation.

	Control (C )				Treatment (T)				*P* values		
	Camel (CC)		Sheep (RC)		Camel (CT)		Sheep (RT)		A	T	A × T
	Mean	SE	Mean	SE	Mean	SE	Mean	SE			
**P: Bacteroidetes**											
F: BS11_gut_group	0.58	0.28	0.11	0.007	0.15	0.03	0.07	0.01	0.14	0.12	0.2
F: RF16_group	0.6^b^	0.14	0.15^a^	0.017	0.7^b^	0.16	0.11^a^	0.016	0.01	0.78	0.49
F: F082	6.8^c^	1.01	1.18^a^	0.49	3.62^b^	0.15	1.17^a^	0.28	0.0011	0.080	0.08
F: Muribaculaceae	1.27	0.07	0.57	0.30	1.07	0.12	1.17	0.37	0.43	0.17	0.028
F: Prevotellaceae	26.9^a^	3.50	21.5^a^	1.01	21.5^a^6	1.59	44.1^b^6	9.33	0.12	0.04	0.06
G: Prevotella	22.3^a^	3.99	20.3^a^7	1.39	16.9^a^	1.60	43.5^b^	9.3	0.05	0.02	0.07
G: RC9_gut_group	15.3^a^	1.80	38.3^b^	9.8	16.5^a^	0.63	16.3^a^	5.7	0.11	0.14	0.11
**P: Firmicutes**											
G: R-7_group	5.5^a^	0.99	13^ab^	4.27	5.5^a^	0.52	11^b^	2.54	0.04	0.03	0.55
G: Butyrivibrio	1.1^b^	0.19	0.12^a^	0.02	0.84^b^	0.08	0.46^a^	0.08	0.001	0.85	0.07
G: Lachnoclostridium	0.65	0.23	0.17	0.01	0.38	0.08	0.00	0.00	ND	ND	ND
G: Anaerovorax	1.3^a^	0.23	0.5^b^	0.16	1.4^a^	0.16	0.4^b^	0.006	0.009	0.9	0.41
G: unclassified Ruminococcaceae	3.3^b^	0.53	1.8^a^	0.39	3.5^b^	0.04	0.9^a^	0.11	0.004	0.37	0.15
G: Papillibacter	3.5	0.09	0.22	0.02	2.44	0.66	0.00	0.00	ND	ND	ND
Ruminococcaceae_NK4A214	2.37	0.79	4.62	1.09	2.61	0.07	3.59	1.53	0.30	0.44	0.25
G: Ruminococcaceae_UCG-005	0.77^b^	0.14	0.8^a^	0.23	1.5^b^	0.02	1.3^ab^	0.19	0.71	0.01	0.46
G: Ruminococcaceae_UCG-014	1.40	0.43	1.42	0.68	3.72	1.30	2.24	0.17	0.4	0.1	0.37
G: Ruminococcus	1.01	0.10	0.83	0.41	1.13	0.30	0.46	0.09	0.24	0.54	0.28
G: Saccharofermentans	2.06^b^	0.12	0.6^a^	0.14	2.25^b^	0.40	0.5^a^	0.06	0.001	0.84	0.66
G: Succiniclasticum	7.5^b^	1.37	0.6^a^	0.35a	5.3^b^	0.26	5.4^b^	1.3	0.04	0.15	0.01
G: Anaerovibrio			0.27						ND	ND	ND
F: Desulfovibrionaceae	0.65	0.26	0.44	0.06	0.75	0.17	0.47	0.23	0.08	0.8	0.89
G: Desulfovibrio	0.63	0.26	0.32	0.07	0.50	0.14	0.40	0.22	0.16	0.9	0.67
G: Succinivibrio	0.062	0.02	0.00	0.00	0.077	0.02	0.035	0.01	ND	ND	ND
**P: Spirochaetes, F: Spirochaetaceae**											
G: Sphaerochaeta	0.64^ab^	0.35	0.35^a^b	0.10	0.97^b^	0.10	0.2^a^	0.03	0.02	0.68	0.37
G: Treponema_2	0.66 ^ab^	0.26	0.5^a^	0.12	0.9 ^b^	0.09	0.2 ^a^	0.14	0.02	0.96	0.26

*ND* Non-determined, *P* phylum, *F* family, *G* genus.

^a,b,c^: Means in the same row with different superscripts differ significantly (*P* < 0.05).

Phylum Firmicutes was not affected by dietary supplementation and animal species. This phylum was further classified into 13 families dominated by Christensenellaceae, Lachnospiraceae, Family_XIII, Ruminococcaceae, and Acidaminococcaceae (Table 8 and Fig. 2). Family Ruminococcaceae was assigned mainly to Unclassified Ruminococcaceae, *Papillibacter*, Ruminococcaceae_NK4A214_group, Ruminococcaceae_UCG-005, Ruminococcaceae_UCG-014, *Ruminococcus*, *Saccharofermentans*. Family Christensenellaceae was assigned mainly to R-7_group. Lachnospiraceae was dominated by *Butyrivibrio*, *Lachnoclostridium*, and AC2044_group. Genus *Anaerovibrio* was found only in the CC group and disappeared from the camel by YA supplementation. Family_XIII was dominated by *Anaerovorax*. Genus *Succiniclasticum* dominated family Acidaminococcaceae, decreased in camel and increased in sheep by YA supplementation (Table 8 and Fig. 2).

Phylum Proteobacteria was affected by animal species. This phylum was dominated by the family Desulfovibrionaceae, which was assigned mainly to the genus *Desulfovibrio*. Phylum Spirochaetes was increased in camels and was dominated by genus *Sphaerochaeta* and *Treponema*. The relative abundances of phylum Cyanobacteria, Planctomycetes, and Tenericutes were increased by YA supplementation. While phylum Kiritimatiellaeota was declined by YA supplementation. Moreover, phylum Fibrobacteres was increased in sheep and declined in camels by YA supplementation; and the opposite trend was found in phylum Lentisphaerae (Table 8 and Fig. 2).

### Pearson correlation analysis

**Figure 3:**
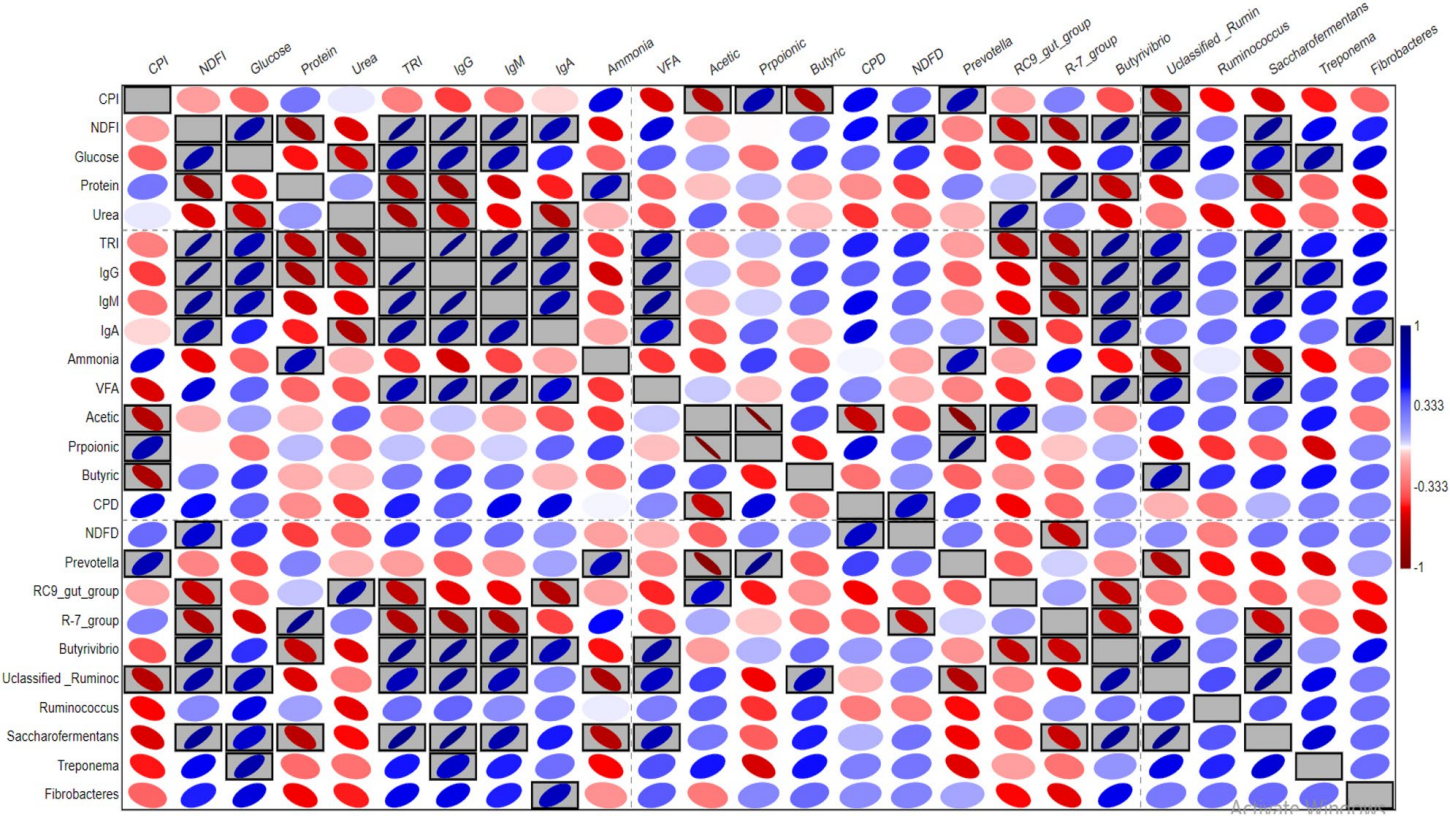
Heatmap based on Pearson correlation analysis. The heatmap shows the correlation relationships between feed intake (NDFI, CPI), blood biochemical and immunological parameters, rumen fermentation parameters, rumen disappearance of CP and NDF, and relative abundances of dominant bacterial genera in the rumen of camels and sheep under investigation. The black boxed ellipses refer to the significant correlations.

Pearson correlation analysis (Fig. 3) was conducted between the relative abundances of dominant bacteria and other parameters. The results showed several positive and negative correlations. For example, NDFI was positively correlated with blood glucose and immunoglobulins, total VFA, NDFD, *Butyrivibrio, Unclassified _Ruminococcaceae, Saccharofermentans,* and *Fibrobacteres.* In addition, NDFI had a negative correlation with blood protein, rumen ammonia, and *R-7_group.*

## Discussion

Using a combination of prebiotic and probiotic is a possible way to stimulate the rumen microbiota to improve rumen fermentation and enhance animal efficiency^31,44^. This study examined the effect of the inclusion a combination of live yeast and algae into the sheep and camels’ diets on feed intake, rumen fermentation, barley straw degradation, blood biochemical and immunology parameters, and rumen microbiota. Animals in this study offered free barley straw that is abundant lignocellulosic residue in desert regions and characterized by high-fiber and low-protein contents^2,5^. The chemical composition of barley straw (Table 1) in the current study was in the line with previous studies^45,46^. Higher NDF content in lignocellulosic biomass represents the main constrain against its digestibility in the rumen^6^. Thus, it is necessary to apply different strategies to enhance the functions of rumen microflora and stimulate fiber fermentation in the rumen^18^. Live yeast and algae were used previously separately in animal feeding, and to the best of our knowledge, there is no information available on the impact of combining these types of algae and yeast on feed utilization and rumen fermentation in ruminant animals. The chemical compositions of microalgae vary according to species, production system, growing condition, and harvesting method^47^. Therefore, different algae species could provide different nutrients and bioactive compounds for animal feeding^48^.

The feed intake of camels and sheep in this study was improved by YA supplementation and was in the range obtained by previous studies on camels and sheep^4,15,49^ (Table 2). The improvement in the feed intake was accompanied by improvement in the rumen disappearance of DM, CP, and NDF of barley straw at 48 h, which is similar to other studies on live yeast and algae^23,50–54^. The improvement in feed intake could be attributed to the increase in the digestibility and higher passage rate due to lipid content in *Spirulina* and *Chlorella*^51,53^, which is supported by the positive correlation between NDFI and NDFD. Furthermore, higher protein disappearance in the rumen of sheep resulted in higher rumen ammonia, which agrees with previous studies^23,55–57^ and that was supported with a positive correlation between CPI and rumen ammonia.

Blood glucose was improved in supplemented camels (TC) and total protein were enhanced in supplemented sheep (TR) (Table 5) by supplementation, which is similar to the previous studies that used live yeast or microalgae^51,58,59^. The enhancement in blood glucose could be explained by the increase in the disappearance of DMD and NDF^51^; this speculation is supported by the positive correlation between blood glucose and NDFD (Fig. 3). The decline of blood urea could be a result of the decrease in protein catabolism and normal kidney function due to YA supplementation^51,60^. The concentrations of blood immunoglobulins, IgA, IgG, and IgM, were increased by YA supplementation, which agrees with other findings on yeast and algae^28,58^. Previous studies indicated that microalgae contain active compounds such as n-3 fatty acids, α-glucan, lutein, and other growth factors that work as immunostimulants^21,51,54^. Also, *Saccharomyces* contain compounds such as β-glucans and oligosaccharides that work as immunomodulators^61^.

It was observed that discrepancies in total VFA production and VFA profile between camel and sheep, which might be attributed to discrepancies in microbial diversity and the relative abundance of bacterial groups^21,62^. The concentration of acetic was declined and propionate was increased in sheep, which was also indicated in previous studies^48,51,53^. Song et al.^63^ included the yeast culture in the diet of growing lambs and observed that rumen pH and total VFA were not affected; rumen ammonia and butyric acid were increased, and acetic acid was decreased. Previous studies^54,57^ revealed that fiber degradation stimulates acetic acid production, which could demonstrate higher acetic acid in supplemented camel group (TC). The increase in the propionic acid production and the decline in the acetic acid could be a positive point for the YA supplementation to reduce the methane production in the rumen as the propionic acid is an alternative hydrogen sink that depresses the methanogensis^64^. The higher bacterial population in supplemented camels (TC) could be attributed to the availability of growth substrates in animal diets^7,56^.

The reduction in total VFA production in supplemented camels (TC) was previously reported by studies that evaluated the microalgae supplementation^48,65^ and yeast/ microalgae combination supplementation (YA) in the horse^21^. Grimm et al.^21^ attributed the reduction in total VFA production after the YA supplementation to the changes in the taxonomy of hemicellulolytic and pectinolytic bacteria such as genus *Prevotella*. This speculation is supported by the decline in the relative abundance of *Prevotella* in supplemented camels (TC). However, investigating the bacterial functions and enzymes is recommended to get a better understanding of the supplementation impact^21^.

The chemical composition of the animal diet and animal species are the main determiner of rumen microbial community structure^8^. YA supplementation and animal species affected the diversity and relative abundances of bacterial communities in the rumen of camels and sheep (Tables 6, 7, 8; Figs. 1, 2). Similar findings were obtained by Song et al.^63^ who investigated the effect of yeast supplementation in the diet of lambs on rumen microbiota. Limited information is available about the effect of the microalgae *Spirulina* and *Chlorella* on rumen microbiota, and few studies quantified some of rumen bacterial genera using qPCR and concluded that algae supplementation affected the copy number of those genera, which supports our results^28,66,67^. The majority of phylum Bacteriodetes was assigned to genus *Prevotella* and RC9_gut_group, reflecting the importance of these genera in rumen fermentation^63^. Genus *Prevotella* is involved in the degradation of different substrates in the rumen, including protein, xylan, pectin, and starch, and can produce propionate^6,7^, which might demonstrate the positive correlation between genus *Prevotella,* propionic acid, and CPD (Fig. 3). Also, the higher relative abundance of this genus and higher propionic in the TR group is explained. Genus RC9_gut_group, within phylum Bacteriodetes, is highly specialized in lignocellulose degradation^7^.

Members of phylum Firmicutes have an important role in fiber degradation in the rumen^7^. This phylum was dominated by Christensenellaceae_R-7_group, *Butyrivibrio, Ruminococcus*, *Saccharofermentans*, *Anaerovorax*, Succiniclasticum (Table 8; Fig. 2), which agree with previous studies^7,63,68–71^. *Christensenellaceae*_R-7_group was also found in the rumen of goats and Yak and has a potential role in cellulose degradation^69,72^. Genus *Anaerovorax* is involved in butyrate production and has a role in ruminal biohydrogenation^68,73^. Additionally, *Butyrivibrio* has proteolytic and fibrolytic activities and is involved in the production of butyrate and ruminal biohydrogenation^6,74^, which could explain the positive correlation between genus *Butyrivibrio* and butyric acid. Genus *Anaerovibrio* was not detected in the rumen of the camels after YA supplementation; also, the relative abundance of genus *Anaerovorax* and *Butyrivibrio* were affected by YA supplementation; these genera are involved in the ruminal biohydrogenation^69,74,75^, which indicate that the biohydrogenation pathway could be regulated by yeast and algae supplementation.

*Papillibacter* genus was previously found in a high proportion in cattle- fed corn stover^76^, indicating that this genus could be involved in fiber digestion^77^. Moreover, genus *Succiniclasticum* has a potential role in fiber degradation^70,78^ and converts succinate to propionate^79^, which could illustrate the increase of this genus and propionic acid in the TR group. Genus *Saccharofermentans* is involved in polysaccharides degradation and produces acetate and propionate^71^, which could support the positive correlation between this genus and NDFD. Unclassified Ruminococcaceae represented a higher proportion of the bacterial community and some of which were increased by YA supplementation; these bacteria might have a role in fiber degradation^6,8,76^. Phylum Spirochaetes was dominated by genus *Treponema* and *Sphaerochaeta,* which were increased by the supplementation. Previous studies^7,80^ indicated that *Treponema* is fiber-associated bacteria, with potential fibrolytic activities. *Sphaerochaeta* is involved in pectin degradation and produces acetate, lactate, and ethanol^81^.

Members of phylum Lentisphaerae are involved in cellobiose fermentation^82^. Phylum Verrucomicrobia was found only in camel and increased by YA supplementation, and has potential role the digestion of plant polysaccharides^7^. Planctomycetes were previously observed in the rumen of the dromedary camel^83^ and have a potential role in cellulose and hemicellulose degradation^84^. Phylum Cyanobacteria that was increased by supplementation are aerobic bacteria capable of scavenging oxygen in the rumen and fermenting carbohydrate during the deficiency of nitrogen^85^. Therefore, this phylum ensures the anaerobic environment to facilitate cellulose degradation by anaerobic microorganisms^86^. Overall, the results showed that the bacterial community was dominated by polysaccharides degrading bacteria, such as*, Prevotella*, *RC9_gut_group*, *Butyrivibrio*, *Ruminococcus*, *Saccharofermentans*, *Christensenellaceae*_R-7_group, and *Treponema, Fibrobacter*^6,69,72,78^. These findings indicate the importance of these genera in the utilization of forage in the rumen. In addition, the relative abundances of some of these genera were affected by YA supplementation, which is in agreement with Ghazanfar et al., and Jiang et al.^61,87^, who reported that yeast promotes the colonization of cellulolytic bacteria to dietary fiber as well as stabilize the rumen pH, which could explain the increase in NDFD at 48 h by YA supplementation.

Rumen microbiota plays a pivotal role in animal immunity^63^. Previous studies^59,61^ mentioned that the improvement in animal immunity could be attributed to the increase in the activity of some cellulolytic bacteria that represent a barrier against pathogens. This trend was observed in the current study, wherever positive correlations between immunity parameters and some cellulolytic bacteria such as *Butyrivibrio*, *Unclassified _Ruminococcaceae*, *Saccharofermentans*, *Treponema*, and *Fibrobacteres* (Fig. 3). Estrada-Angulo, et al.^88^ explained that combining the yeast with prebiotics such as carbohydrates stabilized the rumen pH, modulated the immune response, and improved fiber digestion; besides it inhibited pathogenic bacteria in the gut and alleviated the heat stress. Previous studies^89,90^ that linked animal efficiency with rumen microbiota, showed that efficient cows showed lower methane emission; higher feed intake, milk solid, higher relative abundance of *Prevotella*, *Succinclasticum*; *Treponema*, *Fibrobacter*, *Ruminococcus*; except for milk quality and methane emission that were not studied in this study, all the previous trends were observed in the supplemented animals. These findings highlight the yeast and algae combination as potential promising feed additives. Previous studies showed that live yeast and algae provide a diverse of chemical compounds that are required for the growth of rumen microbiota, such as carotenoids, phycobiliproteins, polysaccharides, lipids, essential fatty acids, n-3 fatty acids peptides, amino acids, and vitamins^50,64^, which encourage the use of yeast and algae combination to supplement the animal diet.

Our results indicated that feed intake, the rumen disappearance of DM, CP, and NDF, and blood immunity were improved in both camels and sheep by YA supplementation. Furthermore, the response of rumen bacteria, rumen fermentation, some of the blood parameters to YA supplementation varied according to animal species. A similar pattern was observed by Lamminen et al.^52^ who noted an individual variation in animals in response to microalgae supplementation. Ghazanfar et al.^61^ concluded that the impact of live yeast on animal performance relies on animal breed, physiological stage, type of animal diet, feeding system, type of yeast, and supplementation dose. It is well known that the anatomy and motility of the camel rumen is different from other domestic ruminant animals^12,14^, and the retention time of ingested feed is longer in the rumen of the camels than in other ruminants, which affects the response to dietary intervention, diet fermentation, the composition of rumen microbiota, metabolism, and blood biochemical parameters^7,14,91,92^.

The study provides insights into the effect of YA supplementation on feed intake and the rumen ecosystem. The results suggest that YA supplementation modulated the rumen microbial community towards increasing degradation of dietary fiber and crude protein, which improved feed intake and affected rumen fermentation parameters. Moreover, blood immunological parameters were improved. The response to YA supplementation varied according to animal species. Combining the microalgae with yeast might be beneficial to animals’ performance.

## Data Availability

All the sequences were deposited to the sequence read archive (SRA) under the accession number: PRJNA767400 Via this link: https://www.ncbi.nlm.nih.gov/sra/PRJNA767400.
